# Relationship Between Elevated Plasma Homocysteine Levels and Severity of Peripheral Neuropathy in Patients With Type 2 Diabetes Mellitus: A Cross-Sectional Analysis

**DOI:** 10.7759/cureus.101955

**Published:** 2026-01-20

**Authors:** Muhammad Toqeer, Masood Uz Zaman Babar, Aiza Bhatti, Shumaila Israr, Eman Masood, Farooq Ahmad Malik, Zunaira Rizwan, Abdul Ahad Mehboob

**Affiliations:** 1 Acute Medicine, Peterborough City Hospital, Peterborough, GBR; 2 Neurology, Isra University, Hyderabad, PAK; 3 Internal Medicine, Al Nafees Medical College, Isra University, Islamabad, PAK; 4 General Medicine, Pakistan Aeronautical Complex Hospital, Kamra, PAK; 5 General Practice, HBS Medical and Dental College, Islamabad, PAK; 6 Biochemistry, Dera Ghazi Khan Medical College, Dera Ghazi Khan, PAK; 7 Cardiology, Abbasi Shaheed Hospital, Karachi, PAK; 8 Pathology, National Laboratory and Testing Centre, Lahore, PAK

**Keywords:** homocysteine, nerve conduction studies, neuropathy severity, peripheral neuropathy, type 2 diabetes mellitus

## Abstract

Background

Peripheral neuropathy is a common and debilitating complication of type 2 diabetes mellitus (T2DM).

Objective

To evaluate the relationship between plasma homocysteine levels and the severity of peripheral neuropathy in patients with T2DM.

Methods

A cross-sectional study was conducted at Sahiwal Teaching Hospital, Sahiwal, from November 2024 to July 2025, involving 325 patients with T2DM. Plasma homocysteine levels were measured, and peripheral neuropathy severity was assessed using the Michigan Neuropathy Screening Instrument (MNSI) and nerve conduction studies (NCS).

Results

Elevated plasma homocysteine levels (>15 μmol/L) were found in 34.5% of patients. The prevalence of neuropathy increased with higher homocysteine levels, with 44.5% of patients with normal homocysteine levels exhibiting neuropathy, compared to 66.7% in those with mild elevation and 85.7% in those with severe elevation. A positive correlation was observed between homocysteine levels and neuropathy severity (r = 0.42, p <0.001). Multivariate regression analysis identified homocysteine as an independent predictor of neuropathy severity (β = 0.38, p<0.001), along with diabetes duration and glycated hemoglobin (HbA1c).

Conclusion

Elevated plasma homocysteine levels are associated with increased severity of peripheral neuropathy in T2DM patients. These findings suggest that homocysteine may serve as a biomarker for identifying T2DM patients at risk for severe neuropathy, with potential implications for early diagnosis and therapeutic interventions.

## Introduction

One of the most common and debilitating complications of type 2 diabetes mellitus (T2DM) is peripheral neuropathy, and the estimates indicate that about 50% of patients with diabetes develop peripheral neuropathy during the life span of their conditions [1]. It is a long-term complication that affects the quality of life of patients, resulting in pain, tingling, numbness, and loss of sensation, especially in the lower limbs. Diabetic peripheral neuropathy (DPN) is a condition that is linked to the severity of the disease, which is linked to a greater risk of foot ulcers, infections, and amputations, which collectively lead to a significant economic burden on healthcare systems at the global level [2]. In addition, DPN is also associated with the presence of other negative consequences, such as falls, impaired mobility, and depression, which makes the work with diabetes even more challenging.

The pathophysiology of diabetic neuropathy is multifactorial, and it is a combination of metabolic, vascular, and inflammatory factors. The neurotoxicity of chronic hyperglycemia is the main cause of nerve damage, which leads to the formation of advanced glycation end products (AGEs) and oxidative stress, which directly harm the endothelial cells of blood vessels, which serve the peripheral nerves [3]. Such compromised blood circulation results in a decrease of oxygen and nutrient uptake to the nerves, which encourages ischemic damage and axonal loss [4]. Moreover, hyperglycemia causes metabolic disorders that affect nerve functioning, especially the metabolism of essential neurotransmitters and other important phases of the cells.

Some recent studies have emphasized how the increase in plasma levels of homocysteine has also been a contributing factor to vascular dysfunction as well as neural damage in diabetic patients and may contribute to the development and progression of DPN [5]. Homocysteine is an amino acid containing sulfur, and it is synthesized as an intermediate in the metabolism of the essential amino acid, methionine. Although homocysteine usually transforms to other metabolites in the body, high levels (hyperhomocysteinemia) may cause endothelial dysfunction, which is a major contributor to diabetic complications. High levels of homocysteine have been found to be involved in the vascular damage, favoring oxidative stress, as well as inflammation, which may eventually lead to compromise of blood flow to the nerves [6]. Besides its vascular effects, it is believed that homocysteine has a nonvascular neurotoxic effect.

Excessive homocysteine can cause neuronal and Schwann cell (which myelinates the peripheral nerves) apoptosis (controlled cell death), which can be a cause of degeneration and demyelination typical of diabetic neuropathy [7]. Moreover, high homocysteine can slow down the activity of enzymes that are important in the well-being of the nerves, including the methionine synthase that plays a role in the production of some significant neurotransmitters such as serotonin and dopamine. This interference with normal neuronal functioning and repair systems can augment the neuropathy symptoms in diabetic patients [8].

Past researchers have reported that there is a positive relationship between high plasma homocysteine and the severity of DPN in T2DM patients. Nonetheless, the results have proven to be mixed, and there is a significant number of studies that have targeted small sample sizes or particular groups of patients, including patients with advanced kidney disease or with severe complications of diabetic disorders. It has been reported that there is a substantial correlation between increased levels of homocysteine and the severity of neuropathy in some studies, and none of the studies have been able to establish a definite relationship between the two [9]. Moreover, various methods of neuropathy evaluation, such as clinical scoring and nerve conduction studies, have given inconsistent outcomes. A more in-depth evaluation of the correlation between homocysteine and the level of DPN severity is also essential, as it might lead to the discovery of the pathophysiology of this prevalent complication of diabetes. The discovery of homocysteine as a possible biomarker of DPN severity may result in the earlier diagnosis and improved risk segregation of T2DM patients [10].

The main objective of the study is to test the hypothesis that elevated plasma homocysteine levels are independently associated with greater severity of diabetic peripheral neuropathy, measured by validated clinical scores and nerve conduction parameters, in patients with type 2 diabetes mellitus, after adjustment for glycemic control and disease duration.

## Materials and methods

A cross-sectional study was conducted at Sahiwal Teaching Hospital, Sahiwal, from November 2024 to July 2025, involving 325 patients with T2DM. The study included adult patients aged 18 years and above who were diagnosed with type 2 diabetes mellitus according to the American Diabetes Association (ADA) criteria. Only individuals with a diabetes duration of at least one year were eligible. Participants were required to exhibit clinical symptoms suggestive of peripheral neuropathy, such as pain, numbness, tingling, or loss of sensation in the lower limbs. In addition, only those who provided written informed consent to participate were enrolled in the study. Patients were excluded if they had type 1 diabetes or gestational diabetes. Individuals with peripheral neuropathy attributable to other causes, including chronic alcohol consumption, chemotherapy, or vitamin B12 deficiency, were also excluded. Those with advanced chronic kidney disease (stages 4-5) or on dialysis were not considered eligible.

Data collection

After obtaining informed consent, demographic and clinical information were collected from all participants. This included age, gender, duration of diabetes, body mass index (BMI), and HbA1c levels to assess glycemic control. Data on comorbid conditions, such as hypertension, dyslipidemia, and cardiovascular disease, were also gathered, as these factors may influence neuropathy severity and homocysteine metabolism. The use of current medications, particularly those affecting homocysteine levels (e.g., folic acid, vitamin B12 supplements) and nerve function (e.g., statins, anticonvulsants), was noted to control for potential confounding factors. Plasma homocysteine levels were measured using high-performance liquid chromatography (HPLC) or an immunoassay method. Fasting venous blood samples were collected after an overnight fast of 8-10 hours. Plasma was separated within 30 minutes using centrifugation at 3000 rpm for 10 minutes. Homocysteine levels were measured using HPLC with fluorescence detection (Agilent 1100 Series, Agilent Technologies, Santa Clara, USA).

In cases where HPLC processing was temporarily unavailable, plasma homocysteine was measured using a standardized chemiluminescent microparticle immunoassay (CMIA) on the Abbott Architect i2000SR platform (Abbott Laboratories, Illinois, USA), following manufacturer-recommended calibration and internal quality control procedures. Patients were categorized as having normal (≤15 µmol/L), mildly elevated (15-30 µmol/L), or severely elevated (>30 µmol/L) homocysteine levels. Participants were classified based on their homocysteine levels: those with levels ≤15 μmol/L were considered to have normal homocysteine, while those with levels >15 μmol/L were categorized into mild (>15 to ≤30 μmol/L) and severe (>30 μmol/L) elevations. Peripheral neuropathy severity was assessed using two primary methods: the Michigan Neuropathy Screening Instrument (MNSI) [11], which includes a self-reported questionnaire and physical examination (monofilament test and ankle reflex), and nerve conduction studies (NCSs) to evaluate the motor and sensory nerve conduction velocities (NCVs) [12] of the lower limbs.

Statistical analysis

Data were analyzed using SPSS version 25.0 (IBM Corp., Armonk, USA). Descriptive statistics were used to summarize baseline characteristics, including mean ± standard deviation (SD) for continuous variables such as age, plasma homocysteine levels, HbA1c, and MNSI scores. Frequencies and percentages were calculated for categorical variables such as gender, comorbidities, and the presence of neuropathy. A p-value ≤ 0.05 was considered statistically significant for all analyses.

## Results

The mean age of participants was 56.8 ± 10.7 years, with a slight male predominance (54.8%). The mean BMI was 28.7 ± 5.1 kg/m², indicating that many participants were overweight. The average duration of diabetes was 8.2 ± 5.3 years, and HbA1c levels averaged 8.5 ± 1.2%, suggesting suboptimal glycemic control across the study population. Hypertension (64.6%) and hyperlipidemia (55.4%) were the most common comorbidities, while metformin was the most commonly used medication (86.1%), followed by insulin (44.6%) (Table 1).

**Table 1:** Detailed Demographics, Clinical Characteristics, and Neuropathy Data by Plasma Homocysteine Levels (n = 325) NCV = Nerve Conduction Velocity; MNSI = Michigan Neuropathy Screening Instrument; HbA1c = Glycated Hemoglobin; BMI = Body Mass Index.

Variable	Normal Homocysteine (<15 μmol/L)	Mild Elevation (15–30 μmol/L)	Severe Elevation (>30 μmol/L)	Total (n = 325)
Age (years), mean ± SD	56.1 ± 10.2	57.9 ± 11.1	59.2 ± 10.3	56.8 ± 10.7
Male, n (%)	115 (54%)	46 (54.8%)	17 (60.7%)	178 (54.8%)
Diabetes Duration (years), mean ± SD	7.4 ± 4.5	8.1 ± 5.2	9.4 ± 5.6	8.2 ± 5.3
HbA1c (%), mean ± SD	8.3 ± 1.1	8.6 ± 1.3	9.1 ± 1.4	8.5 ± 1.2
BMI (kg/m²), mean ± SD	28.5 ± 5.2	28.9 ± 5.3	29.4 ± 4.9	28.7 ± 5.1
Comorbidities, n (%)				
- Hypertension	130 (61%)	58 (69%)	22 (78.6%)	210 (64.6%)
- Hyperlipidemia	115 (54%)	48 (57.1%)	17 (60.7%)	180 (55.4%)
- Cardiovascular Disease	60 (28.2%)	25 (29.8%)	10 (35.7%)	95 (29.2%)
Neuropathy Severity (MNSI Score), mean ± SD	3.8 ± 1.1	5.4 ± 1.3	7.5 ± 1.4	5.2 ± 2.3
Nerve Conduction Velocity (NCV, m/s), mean ± SD	49.2 ± 3.8	45.3 ± 4.5	42.1 ± 5.2	46.2 ± 4.6

Plasma homocysteine levels were elevated in 34.5% of the patients, with 25.8% exhibiting mild elevation (15-30 μmol/L) and 8.6% with severe elevation (>30 μmol/L). The majority of patients (65.5%) had normal homocysteine levels (<15 μmol/L) (Table 2).

**Table 2:** Prevalence of Peripheral Neuropathy by Plasma Homocysteine Levels and Nerve Conduction Study Results Chi-square test was used to find the association between homocysteine category and neuropathy, and one-way ANOVA was used for NCV comparison among groups. MNSI = Michigan Neuropathy Screening Instrument score; NCV = Nerve Conduction Velocity

Homocysteine Level (μmol/L)	Neuropathy Present (MNSI > 3) n (%)	Neuropathy Absent (MNSI ≤ 3) n (%)	p-value (test used)	NCV (m/s), mean ± SD
Normal (<15 μmol/L)	95 (44.5%)	118 (55.5%)	<0.001 (χ² = 24.67)	49.2 ± 3.8
Mild Elevation (15–30 μmol/L)	56 (66.7%)	28 (33.3%)	<0.001 (χ² = 24.67)	45.3 ± 4.5
Severe Elevation (>30 μmol/L)	24 (85.7%)	4 (14.3%)	<0.001 (χ² = 24.67)	42.1 ± 5.2

Among patients with normal homocysteine levels, 44.5% had neuropathy, while this figure increased to 66.7% in those with mild homocysteine elevation and 85.7% in those with severe elevation (Table 3).

**Table 3:** Plasma Homocysteine Levels and Severity of Neuropathy (MNSI Score and Nerve Conduction Velocity) Statistical test used: one-way ANOVA with post-hoc Tukey analysis. MNSI = Michigan Neuropathy Screening Instrument; NCV = Nerve Conduction Velocity; SD = Standard Deviation; ANOVA = Analysis of Variance.

Neuropathy Assessment Method	Normal Homocysteine (<15 μmol/L)	Mild Elevation (15–30 μmol/L)	Severe Elevation (>30 μmol/L)	p-value
MNSI Score, mean ± SD	3.8 ± 1.1	5.4 ± 1.3	7.5 ± 1.4	<0.001 (ANOVA, F = 51.8)
NCV (m/s), mean ± SD	49.2 ± 3.8	45.3 ± 4.5	42.1 ± 5.2	<0.001 (ANOVA, F = 42.3)

Multivariate regression analysis identified elevated plasma homocysteine as a significant independent predictor of neuropathy severity (β = 0.38, p < 0.001), even after adjusting for potential confounders such as diabetes duration, HbA1c, and BMI. Longer duration of diabetes (β = 0.21, p < 0.001) and higher HbA1c (β = 0.10, p = 0.008) were also significant predictors of neuropathy severity. These findings reinforce the idea that poor glycemic control and longer diabetes duration exacerbate neuropathy and that elevated homocysteine independently contributes to neuropathy progression. Age had a small but statistically significant effect on neuropathy severity (β = 0.03, p = 0.04) (Table 4).

**Table 4:** Multivariate Regression Analysis for Predictors of Neuropathy Severity (MNSI Score) β = Standardized Regression Coefficient; CI = Confidence Interval; HbA1c = Glycated Hemoglobin; MNSI = Michigan Neuropathy Screening Instrument.

Predictor	β (95% CI)	Test Statistic (t)	p-value
Plasma Homocysteine (μmol/L)	0.38 (0.22–0.54)	5.22	<0.001
Duration of Diabetes (years)	0.21 (0.15–0.27)	4.47	<0.001
HbA1c (%)	0.10 (0.03–0.17)	2.67	0.008
Age (years)	0.03 (0.01–0.05)	2.09	0.04

A positive correlation (r = 0.42, p < 0.001) was found between plasma homocysteine levels and neuropathy severity as measured by the Michigan Neuropathy Screening Instrument (MNSI). As homocysteine levels rose, MNSI scores, reflecting more severe neuropathy, also increased. Additionally, there was a negative correlation (r = -0.39, p < 0.001) between homocysteine levels and nerve conduction velocity (NCV) (Table 5).

**Table 5:** Prevalence of Neuropathy Based on Clinical and Electrophysiological Assessments MNSI = Michigan Neuropathy Screening Instrument; NCV = Nerve Conduction Velocity; χ² = Chi-square statistic.

Assessment Method	Neuropathy Present (n, %)	Neuropathy Absent (n, %)	p-value
Clinical (MNSI > 3)	175 (53.8%)	150 (46.2%)	<0.001 (χ² = 16.42)
Electrophysiological (NCV < 50 m/s)	167 (51.4%)	158 (48.6%)	<0.001 (χ² = 16.42)

## Discussion

This article examines how high levels of plasma homocysteine and the severity of peripheral neuropathy in type 2 diabetes mellitus (T2DM) patients are related. We have found out that high levels of homocysteine are strongly related to the occurrence of peripheral neuropathy. Namely, 44.5% of the patients whose homocysteine was normal had neuropathy, and this percentage rose to 66.7 and 85.7 in cases of mild and severe elevation, respectively. This tendency indicates the close positive association between neuropathy risk and increased levels of homocysteine. Past studies have also found that higher levels of homocysteine show a positive relationship with a higher risk of developing neuropathy in T2DM, but each of the studies has a different prevalence rate [13]. High levels of homocysteine can facilitate vascular dysfunction, which affects the supply of nerve and increases the severity of neuropathy.

There was a statistically significant correlation between the severity of neuropathy and plasma levels of homocysteine, which positively correlated with the level of homocysteine and MNSI scores (a clinical measure of neuropathy severity) and negatively correlated with nerve conduction velocity (NCV). These results indicate that an increased homocysteine level in the plasma not only correlates with the presence of neuropathy but also with the intensity of neuropathy. It is in agreement with the past studies, which have reported that homocysteine exerted a negative influence on the vascular system and the peripheral nerves to make them incapable of their work, and added to the development of neuropathy [14]. The correlation is found to be negative between homocysteine and nerve conduction velocity and indicates that the higher the level of homocysteine, the slower the nerve conduction, and this is a characteristic of a progressive neuropathy [15].

The importance of glycemic control and duration of diabetes in the prediction of the severity of neuropathy was also emphasized in our study. The duration of diabetes and bad glycemic control (high levels of HbA1c) proved to be important predictors of the neuropathy severity in our multivariate regression. This is consistent with earlier studies, and as per the studies, chronic hyperglycemia has always been known to promote the onset of neuropathy via processes like oxidative stress and endothelial dysfunction [16]. The correlation between homocysteine and the degree of neuropathy was still found to be significant when the factors were controlled, indicating that homocysteine plays an independent role in the development of neuropathy in the T2DM patients.

The result is not new since the previous studies have demonstrated that increased plasma homocysteine levels correlate with clinical and electrophysiological indicators of the severity of neuropathy. High homocysteine can be neurotoxic, i.e., destroy the nerves directly or indirectly, by means of vascular and microvascular pathways that disorganize nerve activity and healing. In addition, the effect of homocysteine on neuropathy can also be through inflammation and oxidative stress, which are known to contribute to the enhancement of nerve injury in diabetic patients [17].

The study provides a compelling case of the need to measure plasma levels of homocysteine in patients with T2DM to detect the high-risk group (T2DM) of developing or developing peripheral neuropathy. Increased homocysteine might also act as a predictive biomarker of neuropathy severity, thus allowing clinicians to act sooner, with more rigorous treatment measures, to prevent additional damage to the nerves. Treatments that have known to reduce homocysteine levels, including the use of folic acid and vitamin B12 supplements, might be considered as possible medical protocols to curb the development of neuropathy since the homocysteine levels are modifiable. These approaches may provide them with more therapeutic advantages to patients of T2DM with high homocysteine levels and neuropathy. Past studies have also shown that the homocysteine-reducing interventions could possibly benefit neuropathy symptoms in individuals with an augmented homocysteine, and additional randomized controlled trials are necessary to assess their clinical usefulness [18,19].

Study limitations

This research study has a number of limitations. To start with, it is cross-sectional, and this makes it difficult to causally conclude between high levels of homocysteine and neuropathy. Longitudinal studies are required to determine a cause-and-effect relationship. Also, nerve conduction studies only record the observable neuropathy, and therefore, subclinical neuropathy might have been missed in a few participants. The possible effects of vitamin supplementation as a part of the neuropathy management strategy involving vitamin supplementation in T2DM patients should also be studied in the future.

## Conclusions

It is concluded that elevated plasma homocysteine levels are significantly associated with both the presence and severity of peripheral neuropathy in patients with type 2 diabetes mellitus (T2DM). The study demonstrates that higher homocysteine levels correlate with worsening neuropathy severity, as assessed by both clinical and electrophysiological measures. Elevated homocysteine is an independent predictor of neuropathy severity, even after adjusting for other important factors such as diabetes duration, glycemic control (HbA1c), and comorbidities. These findings suggest that homocysteine may serve as a useful biomarker for identifying T2DM patients at higher risk of developing or worsening neuropathy. Early recognition of elevated homocysteine levels, along with potential therapeutic interventions such as homocysteine-lowering treatments (e.g., folic acid, vitamin B12), may help mitigate the progression of diabetic neuropathy and improve patient outcomes.
